# The Association between Health System Development and the Burden of Cardiovascular Disease: An Analysis of WHO Country Profiles

**DOI:** 10.1371/journal.pone.0061718

**Published:** 2013-04-18

**Authors:** Yanmei Liu, Koustuv Dalal, Björn Stollenwerk

**Affiliations:** 1 Helmholtz Zentrum München (GmbH), Institute of Health Economics and Health Care Management, Ingolstädter Landstraße, Neuherberg, Germany; 2 Karolinska Institutet, Department of Public Health Sciences, Stockholm, Sweden; 3 Department of Public Health Science, School of Health and Medical Sciences, Örebro University, Örebro, Sweden; Tehran University of Medical Sciences, Iran (Islamic Republic of)

## Abstract

**Background:**

Several risk factors for cardiovascular disease (CVD) have been identified in recent decades. However, the association between the health system and the burden of CVD has not yet been sufficiently researched. The objective of this study was to analyse the association between health system development and the burden of CVD, in particular CVD-related disability-adjusted life–years (DALYs).

**Methods:**

Univariate and multivariate generalized linear mixed models were applied to country-level data collected by the World Bank and World Health Organization. Response variables were the age-standardized CVD mortality and age-standardized CVD DALY rates.

**Results:**

The amount of available health system resources, indicated by total health expenditures per capita, physician density, nurse density, dentistry density, pharmaceutical density and the density of hospital beds, was associated with reduced CVD DALY rates and CVD mortality. However, in the multivariate models, the density of nurses and midwives was positively associated with CVD. High out-of-pocket costs were associated with increased CVD mortality in both univariate and multivariate analyses.

**Conclusion:**

A highly developed health system with a low level of out-of-pocket costs seems to be the most appropriate to reduce the burden of CVD. Furthermore, an efficient balance between human health resources and health technologies is essential.

## Introduction

The health of a population is greatly affected by the health system in operation. The World Health Organization (WHO) defines the health system as all activities that take place to improve, conserve or revitalize health [1]. Health system development varies considerably from country to country. The form of the health system contributes to how much money is spent on health, manages the degree of equality and equity in health care access and regulates how efficiently the available resources are used. However, only a few studies have examined the link between health system characteristics and important health outcomes across nations [2].

The health workforce, which covers all available human health resources, is one important health system component [3]. Not only may the overall amount of available human health resources differ, but also the degree of qualification and the distribution among general practitioners, specialists, nurses, etc. [3]. Health financing is also of great relevance [3]. It might be based on private or statutory health insurance, can be tax based and, furthermore, can consist of a certain degree of out-of-pocket costs. The total amount of health expenditure per capita is also a relevant factor. Considering highly justified interventions, the amount of health expenditure has been judged to be too low in some poor countries [4]. This is accompanied by a considerable degree of out-of-pocket spending, reaching the maximum limit of what private households are able to pay. Health insurance is often limited to the wealthy and to those in formal employment [4].

Cardiovascular disease (CVD), which is defined as a series of disorders of the heart and blood vessels, including heart disease and stroke, is the leading cause of death around the world. It is estimated to be responsible for 30% of all global deaths [5], [6]. For both men and women, over 80% of the world's deaths from CVD take place in low- and middle-income countries (LMCs) [6]. In these countries, many patients cannot afford the high costs of CVD treatment. Owing to high costs, some patients are not willing to pay for CVD treatment in the early stages, which results in even higher payments in the long run [7]. Additionally, in some regions, CVD treatment is not widely available [3].

In contrast, in developed regions, the population is provided with preventive treatment and prompt CVD interventions [8]. As a result, age-standardized mortality from CVD is about three times lower in some developed nations than in some African countries [9]. In developed areas, only 20% of all CVD deaths are in those under 60 years of age, whereas in LMCs, 58% of all CVD deaths occur among the working population [8]. The high incidence of CVD in the working population leads to a large number of premature deaths, which incur great productivity loss and also have a considerable impact on a country's economy [8].

However, the health system may affect the CVD burden of a population. There is a wide range of measures to cure or prevent CVD [10], [11], [12]. The burden of CVD may be reduced by guaranteeing medical treatment to a large proportion of the population, by setting up evidence-based treatment guidelines and by establishing incentives that encourage health professionals to follow these [13], [14]. Additionally, educating the population about risk factors and enhancing early detection are valuable measures [10], [13].

All these measures are expensive, and thus it is expected that the total expenditure on health reduces the burden of CVD. However, as the price levels differ from country to country, one should not only focus on the amount of money spent, but also on the available resources. In particular, these are characterized by the density of nurses, doctors, hospital beds, etc.

However, not all resources that are relevant regarding the burden of CVD can be quantified within a cross-country comparison. For example, based on official statistics, it is hard to judge whether the available resources are concentrated on a small privileged group of people, or distributed more equally among a broad spectrum of the population. However, confounders, such as the dentistry personnel density, can be observed: if resources are focused only on a privileged group of people, the dentistry personnel density is rather low, whereas a high dentistry density may indicate that a broad spectrum of the population may have better access to advanced medical supply.

Understanding the relationship between health system development and the burden of CVD is important to researchers and policy makers. Structuring a health system efficiently may become a further measure, besides medical treatment and lifestyle modification, to reduce the burden of CVD.

Previous studies have mainly focused on selected countries, but hypotheses about the association of health system development and CVD have not yet been tested in a systematic cross-country analysis.

It was intended to use WHO country profiles to test hypotheses concerning health system development on the burden of CVD. The overall objective of this study was to analyse the association between health system development and the burden of CVD, in particular CVD-related disability-adjusted life–years (DALYs).

The research questions were to assess: first, whether the amount of available health system resources is significantly associated with the burden of CVD; second, whether, for a given health budget, out-of-pocket costs are associated with the burden of CVD; and third, whether the distribution of health system resources by resource subgroups (i.e. ‘physician density’, ‘number of hospital beds’, etc.) could partly be related to the burden of CVD.

## Methods

### Data description

Several statistics characterizing the burden of CVD and the health system in the 193 WHO member states have been collected by the World Bank and the WHO (Table 1). All information is based on official organizations in WHO member countries.

**Table 1:** Descriptive statistics characterizing the World Health Organization (WHO) dataset.

	No. of countries (total = 193)	No. of missing values	Mean	Standard deviation	Min	Max
**Variables characterizing the burden of CVD**						
Age-standardized DALY rate due to CVD*	190	3	2988.8	1399.1	843.2	8614.2
Age-standardized CVD deaths*	190	3	349.5	141.3	103.3	832.4
**Health system resources**						
Physicians†	179	14	14.9	14.1	0.5	64
Nurses and midwives†	179	14	36.5	35.0	0.5	163.3
Dentists†	174	19	3.6	3.8	0.5	17.8
Pharmaceutical personnel†	161	32	3.8	3.9	0.5	18.9
Hospital beds†	183	10	31.9	25.8	2	139
Total health expenditure per capita (US$)	193	0	957.1	1286.1	17	7536
**Further health system variables**						
Out-of-pocket health expenditure (% of private health expenditure)	186	7	78.3	20.4	7.3	100

CVD, cardiovascular disease; DALY, disability-adjusted life–year; US, United States.

* per 100,000 inhabitants;

† per 10,000 inhabitants.

The burden of CVD is represented by the age-standardized, CVD-related DALYs per capita, which is also referred to as the CVD DALY rate (a continuous variable with positive values) [15], [16], [17]. DALYs were first introduced by the World Bank in 1993 to measure the global burden of disease [16], [17]. They are the sum of years of life lost and years lived with disability [16], [17]. The data required to calculate CVD DALY rates are very complex and based on numerous data sources [15], [18]. Various assumptions are required, such as how to estimate health-related quality of life and how missing data should be imputed [16], [17], [18], [19], [20].

An alternative estimate, based on fewer assumptions, is the age-standardized CVD mortality rate. Both CVD DALY rates and CVD mortality rates refer to the year 2004 and are based on the WHO standard population [21].

Variables with respect to human health resources are physician density, dentistry personnel density, density of pharmaceutical personnel and density of nurses and midwives (personnel per 10,000 inhabitants, measured on a continuous scale). Estimates are based on administrative reporting systems, household surveys, population censuses and, if none of these sources was available, on health facility assessments. However, the health workforce reference year differs from country to country, ranging from 2000 to 2009 [22], [23].

Further health system variables are the density of hospital beds (2007, per 10,000 inhabitants, measured on a continuous scale), the total expenditure on health (2008, US dollars per capita, measured continuously) and out-of-pocket health expenditure (2005, percentage of private expenditure on health).

The dataset contains a notable proportion of missing values (Table 1), as some statistics were not available for some WHO countries. Variables with the highest proportion of missing values are the density of pharmaceutical personnel (17%) and the dentistry personnel density (10%). Furthermore, there was a wide variation in the ‘total health expenditure per capita’ with a standard deviation more than 30% above the mean. However, this is typical of strongly right-skewed data and appeared to be reasonable [24], [25].

### Statistical analysis

To identify variables that explain the burden of CVD, several generalized linear mixed models (GLMMs) were fitted [24]. Response variables were the age-standardized CVD DALY rate and, as secondary outcome, the age-standardized CVD mortality rate. The WHO region (i.e. Africa, the Americas, South East Asia, Europe, Eastern Mediterranean and Western Pacific) served as random effect. As both response variables are positive and right skewed, gamma-distributed response variables and log-link functions were applied [24], [25]. The model assumptions of the regression models were checked via several residual plots and were approved as being reasonable [25].

Whether the amount of health system resources is associated with the burden of CVD was tested via univariate analyses. In particular, whether the physician density, dentistry personnel density, pharmaceutical personnel density, density of nurses and midwives, density of hospital beds and the total expenditure on health per capita were associated with the burden of CVD was tested. As the corresponding six tests are considered as a multiple test problem, the Bonferroni–Holm correction was applied when drawing a final conclusion [26].

The association between out-of-pocket costs and the burden of CVD for given health system resources was tested via a multivariate regression model. Confounders adjusted for were the physician density, dentistry personal density, density of nurses and midwives, density of hospital beds and the total expenditure on health per capita. The pharmaceutical personnel density was not used for adjustment, as it included a high proportion of missing values. As a secondary outcome, a univariate regression was also performed with out-of-pocket costs as the independent variable.

Whether the distribution of health system resources by resource subgroups could explain parts of the burden of CVD was assessed via an explanatory regression. However, the regression model used for this study question is identical to the confounder model used to answer study question 2 (i.e. the association between out-of-pocket costs and CVD). Conclusions were made based on comparing the regression coefficients of the health resources in the univariate analyses with the regression coefficients in the multivariate regression model.

All analyses were performed with the statistical software package R [27]. A significance level of 5% has been applied, which was a global significance level in the case of the Bonferroni–Holm correction.

## Results

Univariate analyses are reported in Tables 2 and 3. Total health expenditure per capita best explained the variation in DALYs (59% of the deviance), followed by dentistry density (32%). The multivariate regression model was based on 156 WHO member states (81%) and explained 61.0% of the deviance (Table 4). The corresponding multivariate model with response variable ‘age-standardized CVD mortality’ was also based on 156 WHO member states and explained 72.0% of the deviance (Table 5).

**Table 2:** Univariate analyses with response variable ‘age-standardized DALY rate due to CVD’*.

	N	Estimate	SE	p-value	Rate ratio§	Rate ratio 95% CI§	Deviance explained (%)
Hospital beds†	180	−0.0004	0.767	0.63	0.96	[0.74; 1.25]	10.11
**Human health resources**							
Physicians†	176	−0.012	0.003	<0.001	0.28	[0.14; 0.57]	18.49
Nurses and midwives†	176	−0.004	0.001	<0.001	0.61	[0.48; 0.78]	16.68
Dentists†	171	−0.068	0.008	<0.001	0.001	[0.0002; 0.006]	27.39
Pharmaceutical personnel†	158	−0.063	0.010	<0.001	0.002	[0.0003; 0.013]	27.73
**Financial health variables**							
Total health expenditure per capita (US$)	190	−0.0006	0.00002	<0.001	0.97	[0.97; 0.98]	47.11
Out-of-pocket health expenditure (% of private health expenditure)	183	0.006	0.001	<0.001	1.82	[1.33; 2.50]	10.11

SE, standard error; CVD, cardiovascular disease; DALY, disability-adjusted life–year; US, United States.

* per 100,000 inhabitants;

† per 10,000 inhabitants;

§ for 1 additional practitioner per 100 inhabitants, 1 additional hospital bed per 100 inhabitants, additional US$100 health expenditure per capita, additional US$100 out of pocket costs per capita respectively.

**Table 3:** Univariate analyses with response variable ‘age-standardized CVD death rate’*.

	N	Estimate	SE	p-value	Rate ratio§	Rate ratio 95% CI§	Deviance explained (%)
Hospital beds†	180	−0.00007	0.001	0.982	0.99	[0.97; 1.24]	9.94
**Human health resources**							
Physicians†	176	−0.010	0.003	<0.001	0.35	[0.19; 0.65]	18.35
Nurses and midwives†	176	−0.004	0.001	<0.001	0.65	[0.53; 0.80]	17.33
Dentists†	171	−0.058	0.008	<0.001	0.0029	[0.001; 0.014]	31.73
Pharmaceutical personnel†	158	−0.054	0.008	<0,001	0.004	[0.001; 0.02]	30.76
**Financial health variables**							
Total health expenditure per capita (US$)	190	−0.0002	0.00002	<0.001	0.98	[0.97; 0.98]	59.28
Out-of-pocket health expenditure (% of private health expenditure)	183	0.005	0.001	<0.001	1.80	[1.38; 2.36]	18.98

SE, standard error; CVD, cardiovascular disease; DALY, disability-adjusted life–year; US, United States.

* per 100,000 inhabitants;

† per 10,000 inhabitants;

§ for 1 additional practitioner per 100 inhabitants, 1 additional hospital bed per 100 inhabitants, additional US$100 health expenditure per capita, additional US$100 out of pocket costs per capita respectively.

**Table 4:** Multivariate regression model with response variable ‘age-standardized DALY rate due to CVD’*.

	Estimate	SE	Test statistic	p-value	Rate ratio§	Rate ratio 95% CI§
(Intercept)	8.087	0.1317	61.4	<0.001		
**Health system resources**						
Physicians†	0.0008	0.0038	0.20	0.843	1.08	[0.51; 2.30]
Nurses and midwives†	0.0024	0.0012	2.01	0.046	1.28	[1.01; 1.60]
Dentists†	−0.0284	0.0113	−2.51	0.013	0.06	[0.01; 0.54]
Hospital beds†	−0.0006	0.0010	−0.59	0.555	0.94	[0.78; 1.14]
Total health expenditure per capita (US$)	−0.0003	0.00003	−10.4	<0.001	0.97	[0.96; 0.97]
**Further health system variables**						
Out-of-pocket health expenditure (% of private health expenditure)	0.0019	0.0012	1.54	0.127	1.21	[0.95; 1.54]

SE, standard error; CVD, cardiovascular disease; DALY, disability-adjusted life–year; US, United States.

* per 100,000 inhabitants;

† per 10,000 inhabitants;

§ for 1 additional practitioner per 100 inhabitants, 1 additional hospital bed per 100 inhabitants, additional US$100 health expenditure per capita, additional US$100 out of pocket costs per capita respectively.

**Table 5:** Multivariate regression models with response variable ‘age-standardized CVD death rate’*.

	Estimate	SE	Test statistic	p-value	Rate ratio§	Rate ratio 95% CI§
(Intercept)	5.882	0.1183	49.7	<0.001		
**Health system resources**						
Physicians†	0.00003	0.0032	0.012	0.9904	1.003	[0.54; 1.87]
Nurses and midwives†	0.0022	0.0009	2.39	0.0183	1.25	[1.04; 1.50]
Dentists†	−0.0181	0.0091	−1.99	0.0488	0.16	[0.03; 0.97]
Hospital beds†	−0.0003	0.0008	−0.43	0.6693	0.97	[0.83; 1.12]
Total health expenditure per capita (US$)	−0.0003	0.0001	−12.0	<0.001	0.97	[0.97; 0.97]
**Further health system variables**						
Out-of-pocket health expenditure (% of private health expenditure)	0.0021	0.0010	2.13	0.0346	1.23	[1.02; 1.50]

SE, standard error; CVD, cardiovascular disease; DALY, disability-adjusted life–year; US, United States.

* per 100,000 inhabitants;

† per 10,000 inhabitants;

§ for 1 additional practitioner per 100 inhabitants, 1 additional hospital bed per 100 inhabitants, additional US$100 health expenditure per capita, additional US$100 out of pocket costs per capita respectively.

In univariate analyses, all variables representing health system resources were negatively associated with the burden of CVD. However, although most health system resources variables were highly significant, the hospital bed density was not significant with respect to the age-standardized CVD DALY rate (p = 0.63). The multiple tests considering the association with available health system resources were highly significant for both the CVD DALY rate (p<0.001) and CVD mortality (p<0.001).

Out-of-pocket costs were positively associated with the age-standardized CVD DALY rate in univariate analysis (p<0.001) but not significant in multivariate analyses (p = 0.13) (Tables 2 and 4). The health outcome age-standardized CVD mortality gave significant results (p<0.001 for univariate and p = 0.03 for multivariate analysis).

Compared with univariate analyses, some regression coefficients in the multivariate models changed direction. In the multivariate analysis, the physician density (p = 0.84) and the density of nurses and midwives (p = 0.046) were positively associated with age-standardized CVD DALY rates. The dentistry density (p = 0.01) and the total health expenditure per capita (p<0.001) kept their negative association with the burden of disease. The association with hospital beds was not significant (p = 0.56). The response variable age-standardized CVD mortality led to similar results.

## Discussion

In this study, the association between health system development and the burden of CVD was analysed. This has been done by analysing aggregated data referring to WHO member states. Although only a few studies have examined the link between health system strength and health outcomes across nations, several of these have focused on infant mortality as a health outcome [2], [28]. Furthermore, they focused on different health system components.

Although infant mortality is an important health outcome, it does not represent all aspects of the performance of health systems. It is influenced by environmental conditions [29], [30], [31] and does not represent morbidity in an adult population. By focusing on CVD DALY rates, we not only focused on a complementing health outcome, but also adjusted for health-related quality of life. Nevertheless, the results only changed slightly when focusing on the secondary outcome CVD mortality.

Regarding health system components, health financing (i.e. total health expenditure, out-of-pocket costs), human health resources (i.e. the density of physicians, nurses and midwives, dentists and pharmaceutical personnel) and hospital beds were focused on. Others have compared primary care with speciality care [32], national health services with social security systems [28], or focused on specific health care interventions, such as reducing tobacco consumption [8]. Musgrove et al. focused on out-of-pocket spending, social insurance contributions and financing from the government [4]. Most closely related to the analysis presented here, Muldoon et al. performed a cross-sectional study of United Nations member countries [2]. Concordantly, they included human health resources (i.e. physician density and nurse and midwife density) and health financing (total spending per capita, out-of-pocket expenditure, government and private expenditure on health). In addition, they incorporated the corruption perception index, vaccine coverage and access to water and sanitation. Although the last two were relevant predictors with respect to infant mortality, they contribute less to the burden of CVD. However, the current study is based on WHO member states and has used few other relevant variables such as hospital beds.

As expected, a higher level of health care provision, represented by health expenditure per capita and human health resources, was associated with a lower burden of CVD. Only the number of hospital beds was non-significant even though they trended in the same direction.

In contrast, out-of-pocket costs were associated with an increased burden of CVD. However, although this association was significant in univariate analyses, in multivariate analysis, it was only significant regarding CVD mortality, but not regarding CVD DALY rates. High out-of-pocket costs have been discussed and identified previously as a barrier to health care access [2], [33], [34], [35]. However, they have not yet been identified as such based on cross-country comparisons.

In the multivariate regression model, it may appear surprising that nurse and midwife density was positively associated with CVD mortality. This may be explained by the fact that other variables already determine the degree of health provision in the population: total health expenditure per capita approximately defines the amount of available resources. For a given health budget, an increase in nurses and midwives would lead to a decrease in medical products and technologies, such as medical drugs, imaging technology or stents. Furthermore, as there is a reasonable amount of correlation among the health system resources (see appendix), it is not possible to totally distinguish which of the explanatory variables are responsible for the association with the burden of CVD (multicollinearity). Finally, the midwife density might be a potential confounder, as for example, developed countries are associated with a low birth rate [36].

Dentistry density, on the other hand, was negatively associated with the burden of CVD in the multivariate model. This might be surprising, as there seems to be no causal relationship between dentistry treatments and CVD. However, dentistry density may be interpreted as an indicator of how well the basic medical needs of a population are already being fulfilled. A high dentistry density indicates that a high standard of health care is supplied to a large proportion of the population. Therefore, it results in lower CVD mortality.

In the analysis, no adjustment was made for the risk factors for CVD. Although such an adjustment was originally intended, it turned out not to be suitable. There were various reasons: first, effective CVD prevention leads to a higher prevalence of some risk factors within the population, such as a higher proportion of the elderly or a higher proportion of males. To uncover causal relationships, it is not suitable to adjust for variables that are causally affected by the response variables. Second, some risk factors were regarded as highly confounded. For example, female smoking was negatively associated with the burden of CVD (results not shown), whereas smoking is known to be positively associated with CVD [37]. Third, average alcohol consumption was not feasible for adjustment, because it does not give any information about the distribution of alcohol consumption within the population. Whereas previous studies have shown a positive association between CVD and alcohol consumption in heavy drinkers, a protective effect was found in those with moderate alcohol consumption [38], [39]. Fourth, many potential adjustment variables (i.e. CVD risk factors) had a large proportion of missing values. Thus, overall, adjustment for CVD risk factors did not appear to be adequate.

The analysis was based on GLMMs. Compared with ordinary linear regression models, GLMMs have the advantage that they allow the response variable to follow distributions other than the normal distribution [24], [40]. In the current case, an assumed normal distribution would have led to fitted values outside the valid range, which means that the CVD mortality rate and the CVD DALY rate would have yielded negative fitted values for some countries. Applying the gamma distribution paired with the log-link function guaranteed the fitted values to be positive. Although the valid range for mortality rates covers the whole range of positive real numbers, the valid range of DALY rates, on the basis of assuming an upper limit for life expectancy, has an upper bound. However, the ranges of fitted values and of observed CVD DALY rates were similar.

Because of data unavailability, the number of explanatory variables considered was limited. Furthermore, data are based on official statistics from WHO member states and varied with respect to methodology. Another point to consider is that the aggregated data do not represent variation within the population. The number of observations, represented by the 193 WHO countries, is relatively small compared with sample sizes commonly used for multivariate regression analysis [41]. In consequence, some relevant associations might not have been detected. Furthermore, the analysis is based on cross-sectional data. These do not allow the drawing of causal inferences from the results.

The study design was only suitable to detect associations between health system variables, even though causal effects are of much greater interest. The selected health system indices indicate many other social and economic conditions that can influence the burden of CVD. Also, the current condition of CVD is the result of many factors occurring in previous years or decades.

The CVD DALY rates and CVD standardized mortality rates are based on WHO and World Bank estimates for each country. Many of the countries do not have exact and detailed input data for estimating the burden of CVD and thus many assumptions were made. In consequence, the findings may be biased by the hidden assumptions of WHO [16], [17].

One might also question why we did not perform a panel data analysis, as some variables were available for multiple years. However, this was only the case for some of the variables, several of which had a high proportion of missing values. Furthermore, as stated above, the burden of CVD variables was calculated based on multiple assumptions. Their suitability for panel data analyses can thus be questioned.

Further research is needed to find out which other health system components affect the burden of CVD. Furthermore, in this study, the burden of CVD was applied as the health outcome. Other health outcomes may be affected in a different way and could also be researched.

In this analysis, methods of implementation of the findings to improve a population's health were not studied. Thus, research with respect to implementation is needed. This also includes ethical considerations, as implementation would require a more equal distribution of health care access across a population.

In conclusion, a highly developed health system, represented by total health expenditures and human resources, is associated with a reduced burden of CVD. However, for a given level of health care spending, a large amount of out-of-pocket costs is associated with increased CVD mortality. To minimize the burden of CVD, it also seems to be of relevance to balance human resources with competing health technologies.

## Supporting Information

File S1 Supporting information tables. Table S1 List of WHO countries. Table S2 Countries excluded from regression models. Table S3 Correlation matrix of explanatory variables.
(DOCX)
