# Supplementary material for: The Association between Health System Development and the Burden of Cardiovascular Disease: An Analysis of WHO Country Profiles
Source: PLoS One. 2013 Apr 18;8(4):e61718. doi: 10.1371/journal.pone.0061718 (PMC3630133; doi:10.1371/journal.pone.0061718)
Supplement: File S1 — Supporting information tables. Table S1 List of WHO countries. Table S2 Countries excluded from regression models. Table S3 Correlation matrix of explanatory variables. (DOCX) [file pone.0061718.s001.docx]

# Supporting files

The WHO countries are displayed in Table S1. Table S2 shows which countries were excluded from each regression model because of missing values. The correlation matrix of explanatory variables is displayed in Table S3.

Table S1: List of WHO countries

| **ID** | **Country** |
| --- | --- |
| 1 | Afghanistan |
| 2 | Albania |
| 3 | Algeria |
| 4 | Andorra |
| 5 | Angola |
| 6 | Antigua and Barbuda |
| 7 | Argentina |
| 8 | Armenia |
| 9 | Australia |
| 10 | Austria |
| 11 | Azerbaijan |
| 12 | Bahamas |
| 13 | Bahrain |
| 14 | Bangladesh |
| 15 | Barbados |
| 16 | Belarus |
| 17 | Belgium |
| 18 | Belize |
| 19 | Benin |
| 20 | Bhutan |
| 21 | Bolivia (Plurinational State of) |
| 22 | Bosnia and Herzegovina |
| 23 | Botswana |
| 24 | Brazil |
| 25 | Brunei Darussalam |
| 26 | Bulgaria |
| 27 | Burkina Faso |
| 28 | Burundi |
| 29 | Cambodia |
| 30 | Cameroon |
| 31 | Canada |
| 32 | Cape Verde |
| 33 | Central African Republic |
| 34 | Chad |
| 35 | Chile |
| 36 | China |
| 37 | Colombia |
| 38 | Comoros |
| 39 | Congo |
| 40 | Cook Islands |
| 41 | Costa Rica |
| 42 | Côte d’Ivoire |
| 43 | Croatia |
| 44 | Cuba |
| 45 | Cyprus |
| 46 | Czech Republic |
| 47 | Democratic People’s Republic of Korea |
| 48 | Democratic Republic of the Congo |
| 49 | Denmark |
| 50 | Djibouti |
| 51 | Dominica |
| 52 | Dominican Republic |
| 53 | Ecuador |
| 54 | Egypt |
| 55 | El Salvador |
| 56 | Equatorial Guinea |
| 57 | Eritrea |
| 58 | Estonia |
| 59 | Ethiopia |
| 60 | Fiji |
| 61 | Finland |
| 62 | France |
| 63 | Gabon |
| 64 | Gambia |
| 65 | Georgia |
| 66 | Germany |
| 67 | Ghana |
| 68 | Greece |
| 69 | Grenada |
| 70 | Guatemala |
| 71 | Guinea |
| 72 | Guinea-Bissau |
| 73 | Guyana |
| 74 | Haiti |
| 75 | Honduras |
| 76 | Hungary |
| 77 | Iceland |
| 78 | India |
| 79 | Indonesia |
| 80 | Iran (Islamic Republic of) |
| 81 | Iraq |
| 82 | Ireland |
| 83 | Israel |
| 84 | Italy |
| 85 | Jamaica |
| 86 | Japan |
| 87 | Jordan |
| 88 | Kazakhstan |
| 89 | Kenya |
| 90 | Kiribati |
| 91 | Kuwait |
| 92 | Kyrgyzstan |
| 93 | Lao People’s Democratic Republic |
| 94 | Latvia |
| 95 | Lebanon |
| 96 | Lesotho |
| 97 | Liberia |
| 98 | Libyan Arab Jamahiriya |
| 99 | Lithuania |
| 100 | Luxembourg |
| 101 | Madagascar |
| 102 | Malawi |
| 103 | Malaysia |
| 104 | Maldives |
| 105 | Mali |
| 106 | Malta |
| 107 | Marshall Islands |
| 108 | Mauritania |
| 109 | Mauritius |
| 110 | Mexico |
| 111 | Micronesia (Federated States of) |
| 112 | Monaco |
| 113 | Mongolia |
| 114 | Montenegro |
| 115 | Morocco |
| 116 | Mozambique |
| 117 | Myanmar |
| 118 | Namibia |
| 119 | Nauru |
| 120 | Nepal |
| 121 | Netherlands |
| 122 | New Zealand |
| 123 | Nicaragua |
| 124 | Niger |
| 125 | Nigeria |
| 126 | Niue |
| 127 | Norway |
| 128 | Oman |
| 129 | Pakistan |
| 130 | Palau |
| 131 | Panama |
| 132 | Papua New Guinea |
| 133 | Paraguay |
| 134 | Peru |
| 135 | Philippines |
| 136 | Poland |
| 137 | Portugal |
| 138 | Qatar |
| 139 | Republic of Korea |
| 140 | Republic of Moldova |
| 141 | Romania |
| 142 | Russian Federation |
| 143 | Rwanda |
| 144 | Saint Kitts and Nevis |
| 145 | Saint Lucia |
| 146 | Saint Vincent and the Grenadines |
| 147 | Samoa |
| 148 | San Marino |
| 149 | Sao Tome and Principe |
| 150 | Saudi Arabia |
| 151 | Senegal |
| 152 | Serbia |
| 153 | Seychelles |
| 154 | Sierra Leone |
| 155 | Singapore |
| 156 | Slovakia |
| 157 | Slovenia |
| 158 | Solomon Islands |
| 159 | Somalia |
| 160 | South Africa |
| 161 | Spain |
| 162 | Sri Lanka |
| 163 | Sudan |
| 164 | Suriname |
| 165 | Swaziland |
| 166 | Sweden |
| 167 | Switzerland |
| 168 | Syrian Arab Republic |
| 169 | Tajikistan |
| 170 | Thailand |
| 171 | The former Yugoslav Republic of Macedonia |
| 172 | Timor-Leste |
| 173 | Togo |
| 174 | Tonga |
| 175 | Trinidad and Tobago |
| 176 | Tunisia |
| 177 | Turkey |
| 178 | Turkmenistan |
| 179 | Tuvalu |
| 180 | Uganda |
| 181 | Ukraine |
| 182 | United Arab Emirates |
| 183 | United Kingdom |
| 184 | United Republic of Tanzania |
| 185 | United States of America |
| 186 | Uruguay |
| 187 | Uzbekistan |
| 188 | Vanuatu |
| 189 | Venezuela (Bolivarian Republic of) |
| 190 | Viet Nam |
| 191 | Yemen |
| 192 | Zambia |
| 193 | Zimbabwe |

Table S2: Countries excluded from regression models

| **Models** | **Countries excluded due to missing values** |
| --- | --- |
| Multivariate models^§^ | 2, 6, 10, 12, 13, 15, 21, 40, 47, 51, 69, 70, 74, 93, 98, 112, 114, 119, 126, 130, 134, 145, 148, 152, 158, 159, 171, 184, 185, 186, 187 188, 189, 190, 191, 192, 193 |
| Univariate models^§^ |  |
| Hospital beds | 21, 114, 152, 184, 185, 186, 187, 188, 189, 190, 191, 192, 193 |
| Physicians | 2, 6, 10, 12, 13, 15, 21, 51, 69, 70, 74, 112, 114, 134, 145, 148, 152 |
| Nurses and midwives | 2, 6, 10, 12, 13, 15, 21, 51, 69, 70, 74, 112, 114, 134, 145, 148, 152 |
| Dentists | 6, 10, 12, 13, 15, 21, 51, 69, 70, 74, 93, 112, 114, 130, 134, 145, 148, 152, 158, 159 |
| Pharmaceutical personnel | 2, 6, 12, 13, 15, 18, 21, 35, 37, 51, 53, 55, 69, 70, 73, 74, 85, 93, 94, 112, 114, 123, 130, 132, 134, 145, 146, 148, 152, 158, 164, 183, 186, 188, 189 |
| Total health expenditure per capita (US$) | 21, 114, 152 |
| Out-of-pocket health expenditure (% of private health expenditure) | 21, 40, 47, 98, 114, 119, 126, 152, 159, 171 |

^§^ Missing items are independent of regressor used

Table S3: Correlation matrix of explanatory variables

|  | Physicians | Nurses and midwives | Dentists | Pharmace-utical personnel | Out-of-pocket HE | Total HE | Hospital beds |
| --- | --- | --- | --- | --- | --- | --- | --- |
| Physicians | 1 | 0.640 | 0.515 | 0.393 | 0.009 | 0.501 | 0.010 |
| Nurses and midwives |  | 1 | 0.514 | 0.360 | −0.292 | 0.744 | 0.064 |
| Dentists |  |  | 1 | 0.612 | −0.073 | 0.615 | 0.111 |
| Pharmaceutical personnel |  |  |  | 1 | −0.040 | 0.512 | 0.040 |
| Out-of-pocket HE |  |  |  |  | 1 | –0.385 | –0.0002 |
| Total HE |  |  |  |  |  | 1 | −0.011 |
| Hospital beds |  |  |  |  |  |  | 1 |

HE: Health expenditure
